# A Case of Focal Seizures Presented With Recurrent Sweating and Chills

**DOI:** 10.7759/cureus.53139

**Published:** 2024-01-29

**Authors:** Takayuki Ando, Hirohisa Fujikawa

**Affiliations:** 1 Center for General Medicine Education, School of Medicine, Keio University, Tokyo, JPN; 2 Department of Internal Medicine, Suwa Central Hospital, Nagano, JPN

**Keywords:** non-motor seizure, autonomic seizure, paroxysmal symptoms, pilomotor seizure, focal epilepsy

## Abstract

Focal seizures, characterized by excessive electrical excitation in a brain region, present diagnostic challenges due to diverse manifestations, particularly with non-motor symptoms. Here, we present a 69-year-old Japanese woman experiencing unexplained recurrent episodes of sweating, chills, and shivering. Despite exhaustive investigations that identified no abnormalities, her symptoms remained unalleviated by symptomatic treatments. The episodic nature of her presentations subsequently prompted a clinical suspicion of seizures, leading to further neurological evaluations. Magnetic resonance imaging (MRI) of the brain and electroencephalography (EEG) revealed chronic ischemic changes in the cerebral white matter and intermittent sharp and slow wave bursts in the frontal regions. These findings led to a diagnosis of focal seizures manifesting as autonomic symptoms. The patient's symptoms were successfully treated with carbamazepine. This case illustrates the importance of considering non-motor focal seizures in patients with episodic symptoms, even when routine tests show no abnormalities.

## Introduction

Focal seizures are a type of epileptic seizure characterized by excessive electrical excitation localized within a specific area of the brain [1]. Focal seizures originate in one area of the brain and may not always lead to loss of consciousness. They can present with motor, sensory, cognitive, or autonomic symptoms, which can pose diagnostic challenges due to their diverse and often subtle clinical presentations. Therefore, patients may initially present to various medical specialties rather than directly to neurologists. The identification of paroxysmal events as seizures heavily relies on the presentation of seizure semiology. Herein, we report a case of focal seizures characterized by recurrent episodes of sweating, chills, and shivering, which was successfully diagnosed and managed with carbamazepine.

## Case presentation

**Figure 1:**
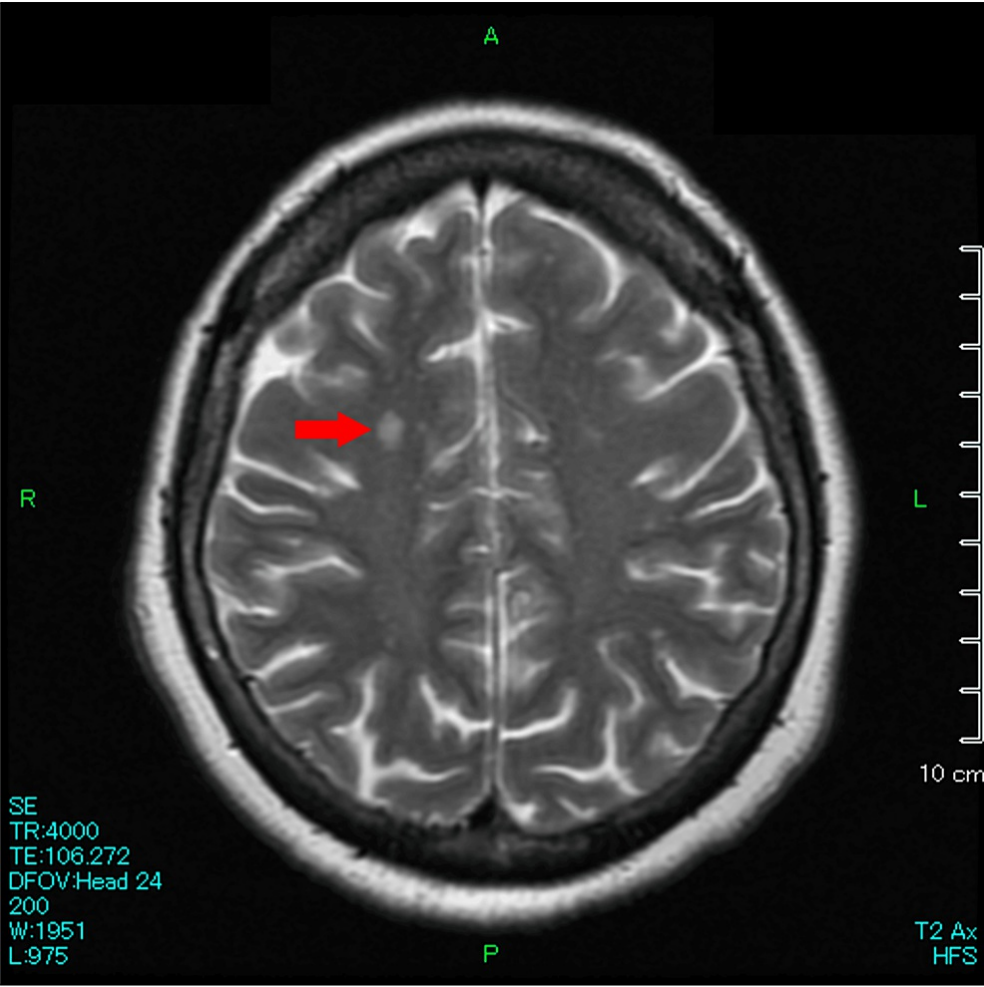
Magnetic resonance image (MRI) of the brain showing T2 hyperintensities in the cerebral white matter.

**Figure 2:**
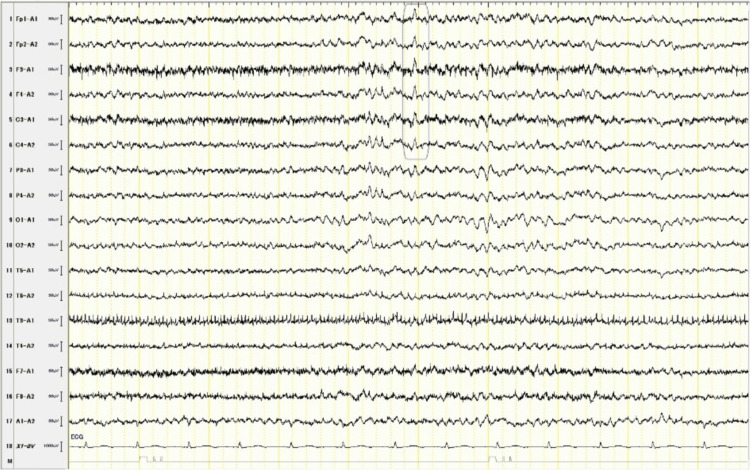
Interictal electroencephalography (EEG) results showing bilateral frontal spikes and slow wave bursts.

A 69-year-old Japanese woman, with hypertension, hyperlipidemia, and post-bilateral total knee arthroplasty, presented with recurring episodes of chills and shivering that began eight months prior. These episodes, characterized by sweating, followed by chills and shivering that resolved naturally within an hour, occurred two to three times a week. Each episode was subsequently followed by profound fatigue, necessitating several hours of rest. On days without these episodes, the patient reported feeling well and was able to carry out her daily activities without limitations. Extensive prior investigations, including hospitalization, failed to reveal etiologies, with blood cultures returning negative and contrast-enhanced trunk computed tomography (CT) scans showing no evidence of abscesses or neoplasms. In the referring hospital, attempts to alleviate her symptoms with herbal medicines and selective serotonin reuptake inhibitors were unsuccessful. Consequently, she was referred to our university hospital eight months following the onset of her symptoms, which had markedly impacted her quality of life. Upon evaluation at our hospital, comprehensive physical and neurological examinations yielded no significant findings, and laboratory tests revealed no abnormalities. The recurrent and paroxysmal presentation of her symptoms heightened the clinical suspicion of focal seizures, prompting further investigations with brain magnetic resonance imaging (MRI) and electroencephalography (EEG). The MRI revealed scattered T2 hyperintensities in the cerebral white matter, indicative of chronic ischemic changes, but there was no evidence of tumors (Figure 1). The EEG showed significant sharp waves and 3-7 Hz slow wave bursts bilaterally in the frontal regions, lasting about two seconds (Figure 2). A diagnosis of focal seizure with a localized cerebral lesion, which pathology was undetermined, manifesting as autonomic seizure, was made, and the patient’s episodes ceased following the commencement of treatment with carbamazepine.

## Discussion

In the present case, a patient with an eight-month history of recurrent shivering and chills was diagnosed with focal seizures. Chills and shivering are manifestations of an elevated set-point in thermoregulation, mediated by vasoconstriction in the skin and the tension of piloerector muscles [2]. While such responses can be triggered by external environmental changes, such as exposure to cold, they are more commonly part of the body’s response to infections [3]. In clinical settings, chills are particularly known as a suggestive sign of bacteremia [4]. However, it is also recognized that chills can result from internal stimuli, such as intense emotions [5]. In the present case, the prolonged course of the symptoms, coupled with normal blood tests, blood cultures, and imaging studies, rendered the likelihood of infection exceedingly low. The absence of symptoms between the episodes of chills and the patient’s otherwise good health pointed to an intermittent nature of the condition, which significantly contributed to the diagnosis. The mnemonic ‘VAPES’ (vascular, allergy, psychiatric, endocrine/electrolytes, seizure/stone/sleep) is suggested to enumerate conditions that present with paroxysmal symptoms [6]. In this patient’s case, based on the history, physical examination, laboratory findings, and imaging tests, the likelihood of other differential diagnoses within the ‘VAPES’ categories, such as moyamoya disease, food allergy, or pheochromocytoma, was considered low. Thus, the focus narrowed to 'Seizures' within the mnemonic, which was a key factor in steering the differential diagnosis toward an epileptic etiology.

The misdiagnosis of epilepsy is a prevalent issue, carrying substantial consequences for affected individuals [7]. Specifically, non-motor focal seizures, which include emotional outbursts and sensory anomalies, are diverse in symptomatology and are more likely to be overlooked in diagnosis compared to motor seizures [8-10]. In fact, the duration to diagnose non-motor focal seizures can be 10 times longer than motor seizures, with a median of 616 days [11]. Thus, focal seizures should be considered in cases of unexplained intermittent symptoms [12,13].

Non-motor symptoms in focal seizures, especially those manifesting as chills, shivers, and piloerection, known as pilo-motor seizures, are exceedingly rare [14,15]. A review of 420 patients with pharmacotherapy-resistant temporal lobe epilepsy found only 16 cases presenting with such seizure forms [15]. The causes varied from hippocampal sclerosis to cavernomas, with about half being cryptogenic. Of these cases, 75% had abnormal brain MRI findings, and 81% showed interictal EEG abnormalities. Single photon emission CT (SPECT) imaging identified abnormalities in 86.7% of those tested. This case, like others, demonstrates the need to consider focal seizures in patients with intermittent symptoms, even when usual tests show no abnormalities. Frequent seizures, as in our case, can lead to detectable interictal EEG abnormalities, and repeated EEG or SPECT imaging can aid in diagnosis.

## Conclusions

This case highlights the essential role of a high index of clinical suspicion in the diagnosis of focal seizures, especially when patients present with atypical autonomic symptoms such as chills and shivering. It also underscores the utility of repeated electrophysiological monitoring and advanced neuroimaging techniques, which can be crucial in uncovering subtle epileptogenic foci that routine examinations may not reveal. Furthermore, this report demonstrates the potential of anticonvulsant therapy to alleviate symptoms and significantly enhance the quality of life for patients afflicted with this form of seizure. It advocates for a thorough consideration of seizures as a differential diagnosis in unexplained paroxysmal symptoms. Ultimately, the case illustrates the heterogeneity of seizure presentations, which can foster earlier recognition and intervention, leading to better patient outcomes.
